# Neurologic phenotypes associated with *COL4A1*/*2* mutations: Expanding the spectrum of disease

**DOI:** 10.1212/WNL.0000000000006567

**Published:** 2018-11-27

**Authors:** Sara Zagaglia, Christina Selch, Jelena Radic Nisevic, Davide Mei, Zuzanna Michalak, Laura Hernandez-Hernandez, S. Krithika, Katharina Vezyroglou, Sophia M. Varadkar, Alexander Pepler, Saskia Biskup, Miguel Leão, Jutta Gärtner, Andreas Merkenschlager, Michaela Jaksch, Rikke S. Møller, Elena Gardella, Britta Schlott Kristiansen, Lars Kjærsgaard Hansen, Maria Stella Vari, Katherine L. Helbig, Sonal Desai, Constance L. Smith-Hicks, Naomi Hino-Fukuyo, Tiina Talvik, Rael Laugesaar, Pilvi Ilves, Katrin Õunap, Ingrid Körber, Till Hartlieb, Manfred Kudernatsch, Peter Winkler, Mareike Schimmel, Anette Hasse, Markus Knuf, Jan Heinemeyer, Christine Makowski, Sondhya Ghedia, Gopinath M. Subramanian, Pasquale Striano, Rhys H. Thomas, Caroline Micallef, Maria Thom, David J. Werring, Gerhard Josef Kluger, J. Helen Cross, Renzo Guerrini, Simona Balestrini, Sanjay M. Sisodiya

**Affiliations:** From the Department of Clinical and Experimental Epilepsy (S.Z., Z.M., L.H.-H., S.K., S. Balestrini, S.M.S.) and Division of Neuropathology (Z.M., M.T.), UCL Institute of Neurology, London, UK; Clinic of Neurology (S.Z.), Department of Experimental and Clinical Medicine, Marche Polytechnic University, Ancona, Italy; Department of Pediatric Neurology and Neurological Rehabilitation (C.S., T.H., P.W., G.J.K.) and Neurosurgery Clinic and Clinic for Epilepsy Surgery (M.K.), Schön Klinik Vogtareuth; Department of Pediatrics (C.S., M.S.), Children's Hospital Augsburg, Germany; UCL Great Ormond Street Institute of Child Health (J.R.N., K.V., S.M.V., J.H.C.), London, UK; Paediatric Neurology and Neurogenetics Unit and Laboratories (D.M., R.G.), A. Meyer Children's Hospital, University of Florence, Italy; Chalfont Centre for Epilepsy (Z.M., L.H.-H., S.K., S. Balestrini, S.M.S.), Chalfont-St-Peter, Buckinghamshire, UK; CeGaT–Center for Genomics and Transcriptomics (A.P., S. Biskup), Tübingen, Germany; Neurogenetics Unit (M.L.), Department of Medical Genetics, Hospital de São João, Porto, Portugal; Department of Pediatrics and Adolescent Medicine (J.G.), University Medical Center Göttingen; Hospital for Children and Adolescents (A.M.), University Clinic Leipzig, Germany; Freiburg Medical Laboratory (M.J.), Dubai; The Danish Epilepsy Centre (R.S.M., E.G.), Dianalund; Institute for Regional Health Services (R.S.M., E.G.), University of Southern Denmark, Odense; Department of Clinical Genetics (B.S.K.), Odense University Hospital; Hans Christian Andersen Children's Hospital (L.K.H.), Odense, Denmark; Pediatric Neurology and Muscular Diseases Unit (M.S.V., P.S.), Department of Neurosciences, Rehabilitation, Ophthalmology, Genetics, and Maternal and Child Health, University of Genoa “G. Gaslini” Institute, Italy; Division of Neurology (K.L.H.), Children's Hospital of Philadelphia, PA; Department of Neurology (S.D., C.L.S.-H.), Division of Neurogenetics, Kennedy Krieger Institute, Baltimore, MD; Center for Genomic Medicine (N.H.-F.), Tohoku University; Department of Pediatrics (N.H.-F.), Tohoku University School of Medicine, Sendai, Japan; Department of Pediatrics (T.T., R.L.) and Institute of Clinical Medicine (K.O.), University of Tartu; Children's Clinic (T.T., R.L.), Department of Radiology (P.I.), and Department of Clinical Genetics, United Laboratories (K.O.), Tartu University Hospital, Estonia; Ludwig-Maximilians-University Munich (I.K.); Department of Pediatric Neurology (A.H.), Clinic Traunstein; Children's Hospital (M.K.), Dr. Horst Schmidt Klinik, Wiesbaden; Altona Children's Hospital (J.H.), Hamburg; Department of Pediatrics (C. Makowski), Technische Universität München, Germany; Department of Clinical Genetics (S.G.), Royal North Shore Hospital, St Leonards; John Hunter Children's Hospital (G.M.S.), New Lambton Heights, New South Wales, Australia; Department of Neurology (R.T.), University Hospital of Wales; Institute of Psychological Medicine and Clinical Neurosciences (R.H.T.), Cardiff University; Division of Neuroradiology (C. Micallef), National Hospital for Neurology and Neurosurgery, London; Department of Brain Repair & Rehabilitation (D.J.W.), Stroke Research Centre, UCL Institute of Neurology, London, UK; Paracelsus Medical University (G.J.K.), Salzburg, Austria; and IRCCS Stella Maris Foundation (R.G.), Pisa, Italy.

## Abstract

**Objective:**

To characterize the neurologic phenotypes associated with *COL4A1/2* mutations and to seek genotype–phenotype correlation.

**Methods:**

We analyzed clinical, EEG, and neuroimaging data of 44 new and 55 previously reported patients with *COL4A1/COL4A2* mutations.

**Results:**

Childhood-onset focal seizures, frequently complicated by status epilepticus and resistance to antiepileptic drugs, was the most common phenotype. EEG typically showed focal epileptiform discharges in the context of other abnormalities, including generalized sharp waves or slowing. In 46.4% of new patients with focal seizures, porencephalic cysts on brain MRI colocalized with the area of the focal epileptiform discharges. In patients with porencephalic cysts, brain MRI frequently also showed extensive white matter abnormalities, consistent with the finding of diffuse cerebral disturbance on EEG. Notably, we also identified a subgroup of patients with epilepsy as their main clinical feature, in which brain MRI showed nonspecific findings, in particular periventricular leukoencephalopathy and ventricular asymmetry. Analysis of 15 pedigrees suggested a worsening of the severity of clinical phenotype in succeeding generations, particularly when maternally inherited. Mutations associated with epilepsy were spread across *COL4A1* and a clear genotype–phenotype correlation did not emerge.

**Conclusion:**

*COL4A1/COL4A2* mutations typically cause a severe neurologic condition and a broader spectrum of milder phenotypes, in which epilepsy is the predominant feature. Early identification of patients carrying *COL4A1/COL4A2* mutations may have important clinical consequences, while for research efforts, omission from large-scale epilepsy sequencing studies of individuals with abnormalities on brain MRI may generate misleading estimates of the genetic contribution to the epilepsies overall.

*COL4A1* and *COL4A2* encode α1 and α2 chains of type IV collagen, respectively, and share a common locus at 13q34. One α2 and 2 α1 chains assemble into a heterotrimer of type IV collagen, a structural component of basement membranes. α-Chains are composed of 3 domains: the amino-terminal region (7S), the carboxy-terminal region (NC1), which initiates heterotrimer assembly, and the collagenous part of the molecule, the triple helix region (THR). The THR is composed of amino acid triplet repeats (Gly-Xaa-Yaa), the first being glycine (Gly) and the other 2 any amino acid.^1^ Most pathogenic *COL4A1/2* mutations are missense and lead to substitution of a glycine with a different amino acid.^2^ In 2005, semi-dominant *Col4a1* mutations were demonstrated to induce perinatal cerebral hemorrhages and predispose to porencephaly in an animal model, with *COL4A1* mutations segregating with human familial porencephaly.^3^ Subsequently, it has been recognized that autosomal dominant *COL4A1* and *COL4A2* mutations cause a broad spectrum of cerebrovascular disease, whose onset occurs from fetal life onward and whose severity may range from small-vessel disease to fatal intraparenchymal hemorrhage.^4–8^ While epilepsy is known to be a clinical feature of porencephaly,^3^ the epilepsy phenotypes associated with mutations in *COL4A1* and *COL4A2* have not yet been detailed. We hypothesized that epilepsy could be a manifestation of disease even in patients in whom porencephaly is not evident and aimed to characterize the phenotypes associated with *COL4A1/COL4A2* mutations, seeking genotype–phenotype correlation.

## Methods

### Standard protocol approvals, registrations, and patient consents

This research was approved by the institutional ethics committees of the participating centers. Informed consent was obtained from all participants, or from parents or legal guardians of minors or individuals with intellectual disability. A bespoke questionnaire was used to collect clinical and genetic data.

Data were collected from published and new patients. Published cases were sought using COL4A1 and COL4A2 as keywords on PubMed/PubMed Gene and selected if they provided sufficient clinical details: 31 articles were reviewed.^8–38^

New patients were gathered through informal links and contact with established consortia (EuroEPINOMICS RES and Deciphering Developmental Disorders). They were included if their variants were considered pathogenic, judged as follows: nonsynonymous, splice-site altering, or truncating changes; present less than 2 times in >120,000 controls in the Genome Aggregation Database (gnomAD) browser and de novo, inherited from an affected parent, or found in affected siblings; or found in patients with MRI findings resembling the previously known *COL4A1/COL4A2* phenotype (e.g., with porencephaly). The following clinical variables were assessed for all new patients: maternal complications during pregnancy, antenatal and perinatal history, neuropsychological delay and cognitive disturbances, and seizure history (age at seizure onset, seizure types, seizure frequency, history of status epilepticus, antiepileptic drug history). Seizures were classified according to the 2017 International League Against Epilepsy (ILAE) classification and terminology.^39^ Drug-resistant epilepsy was defined according to the ILAE Consensus.^40^ Available EEG recordings and brain MRI scans were evaluated. *COL4A1* and *COL4A2* mutations were identified through various methods (table 1, doi.org/10.5061/dryad.gj58t0v). The same data were sought from published cases, though were not always available.

### Statistical analysis

Data were tested for normal distribution. We applied the χ^2^ test to estimate the significance of the differences in perinatal complications and Fisher exact test to assess the significance of differences in prenatal evidence of brain pathology in 2 groups (maternal or paternal inheritance). We applied the Wilcoxon matched-pairs signed-rank test to assess the difference in disease severity across generations in families with established disease. Data were analyzed using Stata/IC 11.1 (StataCorp, College Station, TX).

### Immunohistochemistry

Immunohistochemistry was performed from consented surplus resected tissue from case 1 and compared with 3 control cases (additional methods, doi.org/10.5061/dryad.gj58t0v).

### Data availability

Data not published within the article are available in a public repository (doi.org/10.5061/dryad.gj58t0v) and anonymized data will be shared by request from any qualified investigator.

## Results

### General description of previously published patients

Altogether, 123 patients, from 73 different families, and 69 different mutations (63 *COL4A1* and 6 *COL4A2*) were identified.^8–38^ Epilepsy was reported in 55 patients, all analyzed in this study, associated with 44 different mutations (42 for *COL4A1* and 2 for *COL4A2*).^8–29^ Among published cases with epilepsy, there were 12 of maternal origin, 11 of paternal origin, 8 de novo mutations, and 24 with unknown inheritance. Genetic and clinical details are summarized in data available from Dryad (table 2, doi.org/10.5061/dryad.gj58t0v).

### Demographic characteristics, mode of inheritance, and prenatal and perinatal history in new patients

Data are available from Dryad (table 3a/b, doi.org/10.5061/dryad.gj58t0v). There were 46 new patients (24 male) in 9 countries: Germany (n = 14, 2 from the same family), United Kingdom (n = 12), Italy (n = 10, 5 from the same family), Denmark (n = 3), Australia (n = 2), United States (n = 2), Estonia (n = 1), Japan (n = 1), and Portugal (n = 1). In this cohort, 2 families were included (nos. 33a, 33b, 33c, 33d, and 33e and 23a and 23b), in which at least one participant (nos. 33b and 33d and 23a) had epilepsy. In 2 cases (nos. 26 and 28), epilepsy was not found after evaluation in specialized centers, but these cases were retained in the current study because they carried novel mutations and a compatible neurologic phenotype, described below separately. Two cases were excluded from further analysis due to uncertainty about mutation pathogenicity.

In the final group of 44 new patients, mean age at last follow-up was 9.7 years (SD ± 13.4): 7 patients were adults (mean age 35.6 years; SD ± 15.4) and 37 individuals were children (mean age 4.9 years; SD ± 3.7).

De novo mutations were identified in 24 patients; maternal inheritance was found in 5 patients (including 2 sibling pairs), paternal in 6 (2 of whom were siblings). In one family, the parents tested negative, but both the proband (no. 26) and his sister (not included in the study) carried the same mutation; parental mosaicism is assumed but not proven. In 8 cases, inheritance was unknown.

Natural delivery was reported in 28 patients. Delivery was surgical in 15 patients, due to the following complications: prenatal ventriculomegaly (nos. 4 and 10), severe intrauterine growth retardation (no. 24), polyhydramnios (nos. 26 and 37), fetal arrhythmia (no. 2), intrauterine growth retardation in the other fetus (not included in the cohort) (no. 27), fetal microcephaly and mild renal pelvic dilation (no. 5), placenta previa and intraventricular hemorrhage in utero detected by fetal MRI (no. 17), mild maternal abdominal trauma 3 weeks before due delivery date and subsequent failure to thrive and pathologic cardiotocographic recording (no. 13), hemolysis–elevated liver enzymes–low platelet (HELLP) syndrome (no. 22), prolonged labor (no. 19/a), and suspected hydrocephalus (no. 32); in 2, the reasons were unknown (nos. 20 and 34). Prenatal evidence of vascular cerebral insult was reported in 7 patients (nos. 4, 10, 17, 19/b, 29, 32, and 37). All patients with prenatal evidence of a cerebral vascular event or a prenatal complication requiring surgical delivery developed severe intellectual disability and abnormal neurologic signs.

Maternal complications during pregnancy included gestational diabetes (no. 38), placenta previa (no. 17), bleeding during the first trimester treated with progesterone together with detection of a single umbilical artery (no. 1), and HELLP syndrome (no. 22). None of the mothers with pregnancy complications carried the mutation found in the affected child.

There were 6 late preterm births (nos. 13, 15, 18, 19/a, 20, and 27). Head circumference at birth was known for 20 patients: 15 (nos. 1, 2, 4, 5, 9, 14, 19/a, 20, 21, 22, 24, 25, 29, 31, and 32) had microcephaly.

### Seizure semiology, EEG features, and anatomo-electroclinical correlations

Patients without epilepsy (nos. 23b, 26, 28, 33a, 33c, and 33e) were excluded from this analysis.

Seizure types included focal-onset seizures, epileptic spasms, and generalized tonic-clonic seizures without known focal onset. Mean age at seizure onset was 15.4 (SD ± 26.4) months. Focal-onset seizures, defined by seizure semiology and interictal or ictal EEG findings, occurred in 28/38 patients (73.7%), 10 of whom showed multifocal changes on ictal or interictal EEG. Ictal EEG was available in 5 patients. Video-EEG was not available. Among these 28, impairment of awareness during seizures was described in 13 patients; evolution to bilateral tonic-clonic seizures occurred in 11 patients. Status epilepticus or prolonged seizures (lasting >5 minutes) occurred in 15/38 patients (39.5%) (nos. 1, 2, 3, 9, 10, 13, 19/a, 21, 23a, 29, 30, 31, 32, 34, and 36). Status epilepticus was the presenting symptom in 4 patients (nos. 3, 19a, 23a, and 34). In 18/28 (64.3%) patients with focal seizures, EEG showed diffuse abnormalities (spike-wave activity or generalized slowing) and brain MRI revealed widespread white matter alterations (periventricular leukoencephalopathy, supratentorial white matter loss, and thinning of corpus callosum). In 13/28 patients (46.4%), a porencephalic cyst or a malformation of cortical development localized to the same area as the identified seizure onset zone, with additional widespread white matter abnormalities. In 15/28 (53.6%) patients with focal seizures but no porencephaly, we found diffuse abnormalities on brain MRI, including ventricular enlargement and asymmetry or periventricular leukoencephalopathy and extensive white matter loss (nos. 3, 6, 7, 8, 9, 11, 15, 16, 19a, 19b, 27, 30, 33b, 33d, and 34).

Nine patients had epileptic spasms (nos. 12, 13, 17, 18, 20, 25, 32, 35, and 37). EEG was not available for patients 12 and 32. In the other 7 patients, focal onset of spasms was demonstrated on EEG and in 5 patients (nos. 17, 18, 20, 35, and 37) an association was found between EEG localizing features and a structural abnormality on brain MRI. One patient had generalized tonic-clonic seizures only; EEG was not available and it was not possible to exclude a focal onset (no. 29).

Drug resistance was reported in 24/36 (66.6%); 8 patients (22.2%) had a “good response” to treatment. No single drug stood out for efficacy data (table 3a/b, doi.org/10.5061/dryad.gj58t0v).

Three patients had surgical treatment for epilepsy. One patient (no. 27) with drug-resistant focal seizures underwent corpus callosotomy at 6 years of age, with significant reduction in seizure frequency, with seizures currently every 6–8 weeks.^41^ No complications due to anesthetic or surgery were reported. Patient 24, diagnosed with West syndrome at 6 months, underwent corpus callosotomy at 20 months.^42^ After 1 month of reduced seizure frequency, drug-resistant seizures returned and psychomotor delay became evident. Functional hemispherectomy was then performed, leading to seizure freedom and subsequent improved head control and eye contact. No surgical complications were reported. Patient 1 had surgery to remove a left temporo-occipital dysplasia at 21 months: the pathology is reported below. He remained seizure-free at the latest follow-up, 1 year after surgery.

Of the 55 published patients with reported epilepsy, description of epilepsy phenotypes was provided in 16. Focal seizures were reported in 11 patients: 5 had porencephaly on MRI; 6 had periventricular leukoencephalopathy and irregular enlargement of the lateral ventricles. Four patients with focal epilepsy had EEG records reported, 2 showing a focal abnormality and generalized slowing and spike-wave activity, with extensive hemispheric white matter loss and right-sided porencephalic cyst on MRI. In one patient, EEG showed a slow background and generalized spike-wave discharges, with periventricular leukoencephalopathy and calcifications on MRI. One patient had generalized tonic-clonic seizures and a right-sided porencephalic cyst, 1 had epileptic spasms with good response to vigabatrin and extensive periventricular white matter changes, 1 had epileptic encephalopathy, and 2 had neonatal seizures.

In a subgroup of the new patients (5/38 [13%]) (nos. 3, 7, 8, 33/d, and 34) and 4/55 published cases^12,17,21,28^ (7%), epilepsy was the presenting clinical problem.

### Neuropsychological development and neurologic examination in patients with epilepsy

Intellectual impairment was found in 39/55 previously published cases and in 36/38 new patients.

Neurologic examination showed a wide spectrum of motor abnormalities: pyramidal signs and spasticity were reported in 21 new patients, dystonic features in 7, and hypotonia at birth in 12. Four new patients (2 children [nos. 3 and 34] and 2 adults [nos. 33/d and 8]) had normal neurologic examination at the mean age of 22.7 years at observation (SD ± 18.9 years): notably, patients 8, 34, and 33/d had epilepsy onset after the first year of life, at 11, 5, and 6 years, respectively. In published patients, neurologic examination was abnormal in all but one.

### Extra-CNS involvement in patients with epilepsy

Ocular defects, reported in 16/55 published patients and 19/38 new patients, were the most frequent extra-CNS signs and comprised congenital cataract, retinal vessel tortuosity, and anterior chamber dysgenesis. Increased serum creatine kinase or muscle cramps were documented in 6 new and 7 published patients. Kidney abnormalities (hematuria, hydronephrosis, renal agenesis, and polycystic kidneys) were found in 3 published and 3 new patients. Cardiac disease, reported in 3 new patients and in 2 published patients, comprised mitral valve prolapse, ventricular septal defect, tricuspid regurgitation, and patent foramen ovale. The extra-CNS signs were already present at the time of onset of epilepsy; the timing of onset of the increased serum creatine kinase could not be established from the histories and records available for review (tables 2 and 3a/b, doi.org/10.5061/dryad.gj58t0v).

### Brain MRI findings in patients with epilepsy

**Figure 1:**
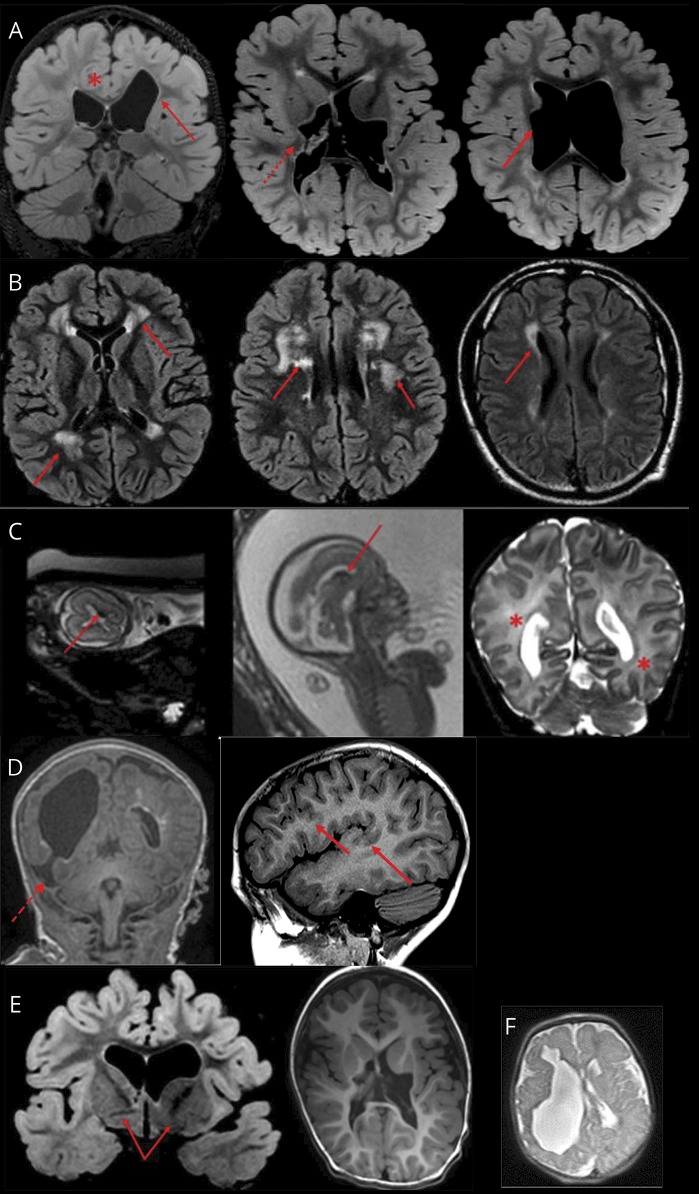
The spectrum of imaging abnormalities observed with *COL4A1* mutations (A) Ventricular enlargement (arrows) and dysmorphism (dotted arrow), thinning of corpus callosum (*), white matter loss (patient 1). (B) Periventricular leukoencephalopathy (arrows) (patient 33/a). (C) Acute germinal matrix hemorrhage on fetal brain MRI (arrows) and consequent extensive leukoencephalopathy on postnatal brain MRI (*) at 8 days of life (patient 33/c). (D) Malformations of cortical development: porencephaly with schizencephalic cleft (dotted lines) and polymicrogyria (arrows) (patient 17). (E) Dysmorphism and asymmetry of basal ganglia (patient 30). (F) Porencephaly (patient 17).

A wide spectrum of abnormalities, summarized in figure 1, was observed on brain MRI. In 29 cases, the brain MRI was performed at epilepsy onset. Porencephaly (figure 1F) was found in 31/55 (56%) published patients and in 15/38 (39.5%) new patients. All patients with porencephaly had a complex syndromic presentation, with severe developmental delay, abnormalities on neurologic examination, and early-onset, drug-resistant seizures. Malformations of cortical development (MCD) (figure 1D), including schizencephaly, polymicrogyria, focal cortical dysplasia, and nodular heterotopia, were identified in 11 new (28.9%) and 7 (11%) published patients. Where present, MCD were always associated with signs of white matter vascular insult (i.e., periventricular leukoencephalopathy, ventricular dysmorphisms, or white matter thinning).

Periventricular leukoencephalopathy (figure 1, B and C) was reported in 11/55 (20%) published and 16/38 (42.1%) new patients. Asymmetry of the lateral ventricles or basal ganglia (figure 1, A and E) was reported in 9/55 (16.4%) published and 22/38 (57.8%) new patients. Posterior fossa abnormalities were reported in 6 new (15.8%) (nos. 2, 9, 17, 18, 29, and 38) and 7 (12.8%) published patients. In one new patient (no. 31), MRI angiography showed reduced development of left medial and posterior cerebral arteries.

Longitudinal MRI data were available only for patients 1, 2, 5, 8, 9, 10, and 16: subsequent MRIs were performed within 3 years from the first one, except for patients 5 and 31, with 5 and 12 years follow-up, respectively. In all cases, consecutive brain MRI findings were stable.

### Phenotypes of patients 26 and 28

Patient 26 (*COL4A1* p. G1169S), aged 17 at last follow-up, had moderate learning difficulties and left hemiparesis. EEG was normal and brain MRI, stable after 2 years, showed bilateral fronto-parietal polymicrogyria and schizencephaly, periventricular nodular heterotopia, and white matter loss.

Patient 28 (*COL4A1* p.G1207V), aged 16 years at last observation, had severe language impairment with dysarthria, language automatism, and left spastic hemiparesis. Brain MRI showed right fronto-parietal schizencephaly. No seizures were reported. He had agenesis of the right kidney and a severe ocular dysmorphism with bilateral ptosis, hemangioma of the left superior eyelid, right cataract, and bilateral retinal atrophy.

### Pathology

**Figure 2:**
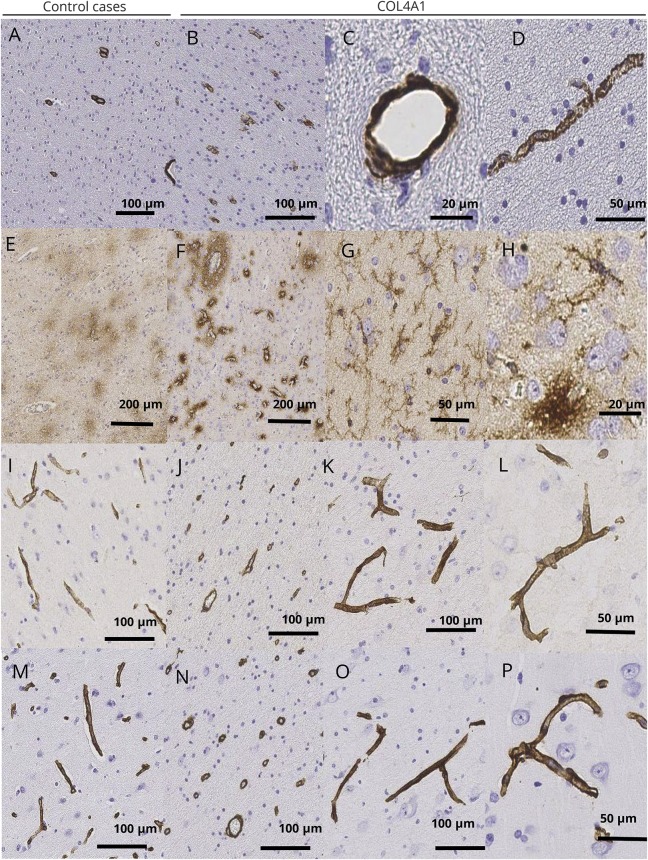
Neuropathologic evaluation of vascular pathology and blood–brain barrier integrity (A–D) Immunostaining with smooth muscle a-actin (SMA) shows no disruption or loss of vascular smooth muscle cells. More numerous SMA-immunopositive blood vessels in white matter were observed in the *COL4A1* case (no. 1) (B) than in the control case (A). SMA expression in the *COL4A1* case was not restricted to vascular arterioles (C) but was also observed in numerous vascular capillaries (D). (E–H) Evaluation of blood–brain barrier integrity using immunoglobulin G (IgG) immunostaining. More marked IgG–immunopositive small vessel permeability was observed in the *COL4A1* case (F) than in the control case (E). Strong IgG immunolabeling was observed in processes with glial morphology, but not in neurons in the *COL4A1* case (G, H). (I–P) Integrity of basal membrane. (I–L) Laminin immunolabeling was present in arterioles and in capillaries with homogenous thickness in both the control (I) and the *COL4A1* case (J–L). (M–P) Expression of the COL4A1 protein. A regular pattern of immunolabelling in arterioles and capillaries presenting homogenous thickness was observed in the control case (M) and the *COL4A1* case (N–P). Scale bar (A, B, I–K, M–O) = 100 µm; (C, H) = 20 µm; (D, G, L, P) = 50 µm; (E, F) = 200 µm.

Pathology results are detailed in figure 2.

### Genetic findings

**Figure 3:**
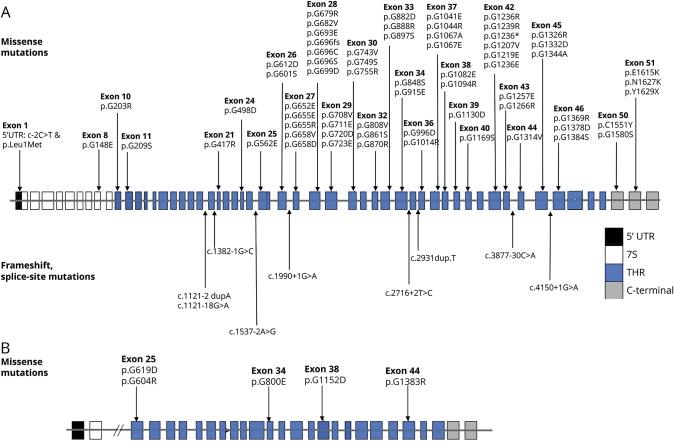
The distribution of mutations in the genes The upper half of each figure depicts missense mutations, the lower half frameshift and splice-site mutations. (A) Distribution of *COL4A1* mutations. (B) Distribution of *COL4A2* mutations.

Seventy-three *COL4A1* mutations, 42 from published and 31 from new patients, and 5 *COL4A2* mutations, 2 from published and 3 from new patients, all associated with epilepsy, and the 2 novel mutations of cases 26 and 28 are shown in figure 3, A and B. In the new cohort, 31 novel mutations were identified. *COL4A1* (NM_001845) mutations were spread across the whole gene: 2 mutations were in the transcription initiation site, 68 in the THR, and 5 localized to the C-terminal region. The 2 mutations localized in the initial part of the gene (nos. 1 and 2) were associated with a severe clinical phenotype, with onset of epilepsy at 3 months and a history of status epilepticus. THR mutations comprised 9 splice-site and frameshift mutations, 1 substitution leading to protein truncation, and 58 missense mutations leading to glycine substitutions in Gly-Xaa-Yaa motifs. No obvious correlation between the position of the mutation and the severity of the associated phenotype was observed in the THR region. The 5 mutations in the C-terminal domain were all missense and were all associated with a severe syndromic picture, except the variant p.C1551Y, found in patient 34, with focal epilepsy and behavioral problems, normal neurologic examination, nonspecific white matter lesions, and an arachnoid cyst on MRI.

The *COL4A1* p. G601S variant is newly described, identified in 2 new patients (nos. 8 and 9). Patient 8 had developmental delay, moderate cognitive impairment, autism, and normal neurologic examination. Focal-onset drug-resistant seizures started at 11 years of age. Brain MRI showed extensive supratentorial white matter loss and abnormalities, originally interpreted as perinatal infection. Patient 9 had onset of focal drug-resistant seizures at age 10 months; neurologic examination showed microcephaly and hypotonia at birth. MRI showed periventricular white matter loss, thinning of the corpus callosum, and cerebellar atrophy.

The *COL4A1* p.G720D variant was previously described in 2 families. In the first family,^30^ 5 individuals had malformations of the anterior chamber of the eye and cerebral vasculopathy (one member had infantile-onset hemiparesis). No epilepsy was reported in this family. In the second family,^24^ 2 members were affected. The proband had intraventricular hemorrhage resulting in porencephaly and developed “generalized epilepsy” in the first year of life. He also had optic coloboma and cataract. His father had bilateral congenital cataracts, migraine, and recurrent TIA.

We found 5 recurrent *COL4A1* mutations: p.G601S, p.G720D, p.G749S, p.G1044R, and p.G1239R. As detailed below, they were usually associated with phenotypic variability.

The *COL4A1* p.G1044R variant was described as a de novo mutation in a patient with low birthweight, congenital bilateral cataracts, microcephaly, and porencephaly.^19^ Among the new patients, we found a similar phenotype in a child who died at 6 years of age (no. 24) and had bilateral porencephaly, intractable epilepsy, profound global developmental delay, microphthalmia, and congenital cataracts.

The *COL4A1* p.G749S variant was described in an Italian family^11^: 2 siblings had spastic quadriparesis and focal epilepsy; their father had normal intellect and mild left hemiparesis. The same variant was found in a patient with prenatal ultrasound evidence of massive brain parenchymal hemorrhage and neonatal seizures.^27^ His father, who had the mutation, only had minor white matter abnormalities on brain MRI.

The *COL4A1* p.G1239R variant was first reported as a paternally inherited mutation in a child with intracranial hemorrhage identified on prenatal screening and subsequent left porencephaly and progressive hemolytic anemia.^23^ The father had features of hereditary angiopathy with nephropathy, aneurysms, and muscle cramps (HANAC) syndrome. In our series of new cases, the same mutation was found de novo in an affected 3-year-old girl. Surgical delivery was performed because prenatal hydrocephalus was suspected. The child developed microcephaly, severe cognitive impairment, and drug-resistant epileptic spasms. Of note, her paternal grandmother died of a ruptured cerebral aneurysm (no other clinical details were known).

The 5 *COL4A2* (NM_001846) mutations were all missense mutations and localized to the THR domain.

### Analysis of pedigrees

**Figure 4:**
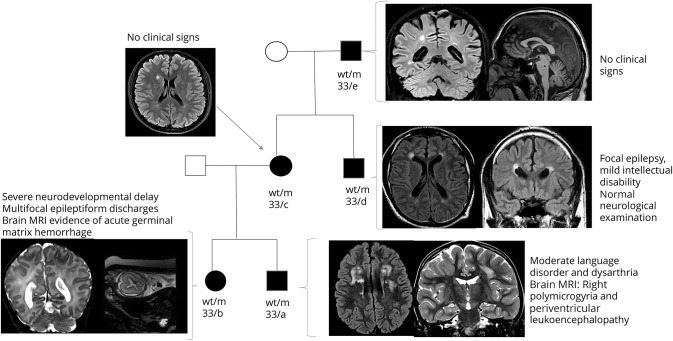
MRI findings in a pedigree (cases 33a–33e) with *COL4A1* mutation (p.G1369R) wt/m = wild-type/mutated.

The pedigree of an Italian family from the new group is presented in figure 4. Five members carried mutation *COL4A1* p. G1369R and presented with very varied clinical phenotypes.

Fifteen previously published kindreds were analyzed and are illustrated in data available from Dryad (figure e-1, doi.org/10.5061/dryad.gj58t0v). Severe phenotypes were preceded by less typical clinical presentations of disease in the previous generations. This generational gradient of disease severity was associated with maternal inheritance in 11 families and paternal inheritance in 4.

To assess the severity of disease between different generations in these families with established mutation, we built an additive score including neurologic (epilepsy; intellectual impairment; abnormal neurologic examination = 1 point each) and neuroimaging (porencephaly; brain hemorrhage; diffuse leukoencephalopathy; asymmetric ventricular system = 1 point each) data. The score was calculated for patients and relatives in each family, and a significant difference in disease severity was found when comparing across each generation pair (Wilcoxon matched-pair signed-rank test, *p* < 0.001). There was no significant difference between the groups of maternal and paternal inheritance for the number of perinatal complications (χ^2^, *p* = 0.07) or prenatal evidence of brain pathology (Fisher exact test, *p* = 0.68). In the first 4 pedigrees (figure e-1, a–d, doi.org/10.5061/dryad.gj58t0v), patients with severe phenotypes, including porencephaly, succeed less severe phenotypes having epilepsy as their main clinical feature. Of note, in the less severely affected patients, brain MRI showed nonspecific findings (in particular periventricular leukoencephalopathy and asymmetric enlargement of the ventricular system) that would not have suggested the genetic diagnosis until a more typical and severe phenotype appeared in the family.

## Discussion

In our series of new and published patients, the neurologic patterns associated with *COL4A1/COL4A2* mutations comprised a typical severe presentation and a spectrum of less common phenotypes, in which epilepsy can be the predominant feature. In the present study, we retained the term “porencephaly,” notwithstanding its lack of specificity, as it is in common clinical usage. The typical severe phenotype was characterized by porencephalic cysts on brain MRI, and clinically defined by severe developmental delay, intellectual and behavioral difficulties, microcephaly, and motor abnormalities on neurologic examination, with involvement of both pyramidal and extrapyramidal systems.

We identified a subgroup of new and published patients in which epilepsy was the main feature leading to medical attention, associated with mild to moderate intellectual or behavioral difficulties, while neurologic examination showed slight and insidiously developing motor abnormalities. Two adult patients with epilepsy had normal neurologic examination: patient 33/d was diagnosed after a severe phenotype of disease appeared in the family. Patient 8 was diagnosed 25 years after epilepsy onset after specialist review. Milder presentations are likely underdiagnosed, due to limited awareness of the full spectrum of neurologic presentations, such that clinicians (in particular those seeing adults) may suspect *COL4A1/2* etiology only in the most severe cases. The diagnosis in milder cases with new-onset epilepsy is challenging because of the nonspecific brain MRI features (i.e., asymmetric ventricular enlargement, diffuse periventricular leukoencephalopathy, white matter thinning), whose causation is frequently attributed to traumatic or hypoxic-ischemic birth injury and intrauterine infections. For instance, new patient 8 was initially diagnosed with epilepsy secondary to unidentified perinatal infection. A similar misdiagnosis was previously described.^22^ Notably, the involvement of other organs (especially eyes, kidneys, and muscles) was found to be already present at the time of onset of epilepsy and can provide a diagnostic clue.

We also observed a generational gradient of disease severity, especially with maternal inheritance. *COL4A1/2* mutations in the fetus induce susceptibility to intrauterine environment stressors that increase the risk of intraventricular hemorrhage.^7^ Since *COL4A1/COL4A2* are among the maternal susceptibility genes for preeclampsia,^43^ we hypothesized that a mutation expressed in the maternal uterus may further increase the risk of prenatal brain complications and, consequently, the severity of disease. Our analysis did not show a significant difference in prenatal and perinatal complications between maternally and paternally inherited cases. However, we suggest this hypothesis as one explanation, for testing in future studies as the low numbers and potential selection biases may have limited the conclusions of the present study. The pedigree in figure 4 also highlights that asymptomatic carriers (nos. 33/c and 33/e) can precede severe phenotypes. This observation suggests a reduced penetrance of *COL4A1/COL4A2* mutations that may partly contribute to the generational gradient of disease severity.

*COL4A1/COL4A2* mutation-related seizures typically had focal onset, also in cases with epileptic spasms. EEG recordings showed focal or multifocal epileptiform discharges and generalized, frequently asymmetric, abnormalities (generalized spike-waves or diffuse slowing). Focal epileptiform discharges were related to a specific lesion (in particular porencephalic cysts, schizencephaly, or polymicrogyria) in 46.4% of patients, while in the remaining patients, less specific EEG abnormalities were described, without a clear correlation with a focal lesion. This complex anatomo-electroclinical picture suggests different pathogenic associations. The most typical is through predisposition to hemorrhagic and ischemic insults, as demonstrated by mouse models.^3,4^ MCD were also associated with *Col4a1* mutations, as a result of defects of cortical lamination.^44^ Here, we found a notably high prevalence (28.9% of new cases) of polymicrogyria, schizencephaly, or focal cortical dysplasia.

De novo mutations seemed more common in the newly identified patients. One possible explanation of this discrepancy is that growing evidence of de novo variants in epilepsy causation has led to an increasing search for such variants, and a slight move away from familial studies. However, the numbers in this study are modest overall and for some published cases data on inheritance were unavailable; thus, we cannot draw secure conclusions on this aspect.

An increased awareness of *COL4A1/2*-related epilepsy phenotypes has clinical and research implications. One is for follow-up. *COL4A1/COL4A2* mutations are established monogenic causes of stroke and can present for the first time in adult life with features of cerebral small-vessel disease, including subcortical hemorrhage and ischemic stroke, with lacunar infarcts, leukoaraiosis, and cerebral microbleeds on MRI,^6^ suggesting a dynamic evolution of *COL4A1/2* leukoencephalopathy. Although our subset of longitudinal MRI data did not demonstrate progressive increase in the burden of cerebrovascular disease, important limitations (young age, low numbers, short follow-up) hamper definitive statements. Although data on the risk from *COL4A1/2* mutations for future intracerebral hemorrhage or ischemic stroke remain limited, these mutations might increase the intracranial hemorrhagic risk in anticoagulated patients: one patient carrying *COL4A1* mutation p.G562E died at age 40 after a spontaneous cerebral hemorrhage while on oral anticoagulation.^25,26^ The intracranial bleeding risk during IV thrombolysis in patients carrying *COL4A1/COL4A2* mutations also needs consideration. The presence of cerebral microbleeds on brain MRI might help to identify those with *COL4A1/COL4A2* mutations at highest risk of intracranial hemorrhage prior to anticoagulation or thrombolysis.^45,46^

Epilepsy surgery, including both functional surgical procedures (like corpus callosotomy) and focal resections, was performed in 3 patients. To our knowledge, patient 1 is the first with a known *COL4A1* mutation to have undergone a resection of MCD, resulting in complete seizure control. Although we are aware of only 3 patients with this genetic condition treated surgically, notably the outcomes have been successful, in terms of both safety and effectiveness. There is rising interest in the role of genetic diagnostics during presurgical evaluation.^47^ The genetic epilepsies are heterogeneous and for some (e.g., focal cortical dysplasia due to mutations in MTOR pathway genes), surgery may be appropriate, while for other genetic conditions surgery may not be effective.^47^ It is therefore desirable that each causation is considered gene by gene in a multidisciplinary team, with, wherever possible, decisions based on understanding of the underlying mechanisms of disease. The evidence so far, although limited, suggests that surgery may be a valid option for drug-resistant *COL4A1/2*-associated epilepsy. The presurgical evaluation should consider other organ involvement (which may contribute to an increased perioperative risk). Broadening the spectrum of clinical phenotypes associated with *COL4A1/COL4A2* mutations may help our understanding of the genetic architecture of the epilepsies. Many large-scale genetic research efforts tend to exclude people with structural changes on MRI, including cysts and periventricular leukoencephalopathy. The epilepsy phenotypes associated with *COL4A1/COL4A2* mutations suggest that this may not be the most comprehensive strategy to determine the full effect of genetic variation in the causation and biology of the epilepsies, or to best apply genetically driven precision medicine approaches.^48^

There are certain phenotypic pointers to considering *COL4A1/2* mutations in individual patients, with implications for individual patient management and for our understanding of epilepsy genetics.

## Publication history

Received by *Neurology* January 12, 2018. Accepted in final form August 17, 2018.

## Glossary

Gly: glycine
HELLP: hemolysis–elevated liver enzymes–low platelet
ILAE: International League Against Epilepsy
MCD: malformations of cortical development
THR: triple helix region
